# Effects of smoking on the retina of patients with dry age-related macular degeneration by optical coherence tomography angiography

**DOI:** 10.1186/s12886-022-02525-5

**Published:** 2022-07-22

**Authors:** Weizhou Yang, Chunyuan Song, Meng Gao, Shuna Wang, Haonan Yu, Yan Li

**Affiliations:** Eye centre, Affiliated Hospital of Weifang Medical University, Kuiwen District, No. 288 Shengli East Street, Weifang, 261035 China

**Keywords:** Optical coherence tomography angiography, Dry age-related macular degeneration, Smoking, Retina, Retinal vessel density

## Abstract

**Background:**

The macula of the retina is analysed using optical coherence tomography angiography (OCTA) to provide clinical basis and explain the mechanism of smoking as a risk factor in dry age-related macular degeneration (AMD).

**Methods:**

This cross-sectional study included 49 normal control nonsmokers, 12 normal control smokers, 38 dry AMD nonsmokers and 35 dry AMD smokers. The foveal avascular zone (FAZ), foveal density (FD) in a 300 μm region around FAZ, vessel densities of the superficial (SCP) and deep (DCP) capillary plexuses and central fovea retinal thickness (FRT) were compared using OCTA. The bivariate correlation analysis was used to evaluate the effect of pack–year history on retina-related indices.

**Results:**

The vessel densities of whole, foveal and parafoveal of SCP and whole and parafoveal of DCP in the control nonsmoking group were all significantly higher than those in the dry AMD nonsmoking group (all *P* < 0.05), whereas the whole vessel density of SCP in the normal smoking group was higher than that in the dry AMD smoking group (*P* = 0.04). The thickness values of the inner and full-layer FRT in the normal nonsmoking group were significantly thicker than those in the dry AMD nonsmoking group (all *P* < 0.01). The pack–year history was negatively correlated with the parafoveal vessel density of DCP (*r* =  − 0.224, *P* < 0.01).

**Conclusions:**

FD, SCP, DCP and FRT are sensitive indices for the detection of early and intermediate dry AMD. DCP is a sensitive indicator that reflects the effects of smoking on the retina. Considerable changes are observed in retinal vessels, suggesting that dry AMD may affect the retinal tissue to a certain extent.

## Background

Age-related macular degeneration (AMD) leads to the irreversible loss of central vision and remarkably reduces the quality of life of patients. For developed countries, AMD is one of the most common causes of blindness amongst the elderly [1]. With an aging population, AMD is becoming an increasingly important and prevalent disease worldwide.

AMD has complex multifactorial pathogenesis [2]. The retinal pigment epithelium (RPE) is a blood–retinal barrier with a variety of functions. Genetic susceptibility, aging, oxidative damage and inflammation can destroy the reciprocal dependence amongst RPE, Bruch membranes and choroidal capillaries and participate in the development of drusen and pigmentary abnormalities at the RPE level [3, 4]. It is classified into two main types: dry (also known as “nonexudative”) and wet (“exudative”). Dry AMD is characterized by drusen, RPE changes, and, in advanced forms, geographic atrophy (GA) — confluent areas of regressed drusen and RPE atrophy. Dry AMD accounts for 85–90% of all AMD cases [5]. Wet AMD is characterized by the development of neovascularization under the retina or RPE that results in leakage of fluid and hemorrhage in the intraretinal, subretinal, or sub-RPE space. Nowadays, the treatments of wet AMD have made great progress, such as macular photocoagulation, photodynamic therapy and anti-vascular endothelial growth factor (VEGF) drugs. However, there is currently no effective treatment for dry AMD. As an important modifiable risk factor, smoking is closely related to AMD. It has increased risk two-times for developing late AMD [6]. Therefore, advocating smoking cessation is essential for dry AMD’s preventive treatment. In recent years, the hot issues in the study of AMD are predominantly focused on the changes in choroid. Vascular changes within the retina appear to be evident in AMD with reports of increased pulsatility and decreased blood flow velocity in the retinal arteries of AMD eyes [7] as well as dilation and attenuation of retinal arteriolar and venular width in early AMD [8–10]. Recent work by using OCTA has also attempted to describe abnormal retinal vasculature in AMD, but exact findings have been inconsistent. 

OCTA can realise the automatic stratified imaging of retinal vessels and shows unique advantages in displaying the blood vessels and flow of each retinal layer [11]. Smoking is one of the common variable risk factors of AMD. Moschos MM et al. [12] analysed 31 smokers and 25 nonsmokers and found that smoking seems to lead to the thinning of the retina. At present, most studies focused on using OCTA to observe the retina and choroid of patients with dry AMD or smokers but lacked the systematic observation of the retina of smoking patients with early and intermediate dry AMD.

In this study, we have used OCTA to observe foveal avascular zone (FAZ), foveal density (FD) in a 300 μm region around FAZ, vessel densities of the superficial (SCP) and deep (DCP) capillary plexuses and inner and outer and full-layer of central fovea retinal thickness (FRT). We have analysed the effect of smoking on retinal changes in dry AMD and explored the possible role of retinal capillaries in the pathogenesis of dry AMD to provide clinical basis on the mechanism of smoking as a risk factor in AMD.

## Methods

This prospective cross-sectional study adhered to the tenets of the Declaration of Helsinki and was approved by the Research Ethics Committee of the Affiliated Hospital of Weifang Medical University (Approval No. wyfy-2020-ky-154). All subjects signed the informed consent voluntarily.

### Patients

A total of 73 patients with early and intermediate dry AMD (73 eyes) and 61 age-matched normal controls (61 eyes) were recruited from our department from May 2020 to January 2021. In accordance with smoking history, subjects were further divided into nonsmoking and smoking groups. The standard pack–year method was used to measure the incidence and degree of smoking, and participants with at least five years of smoking history and at least one pack of cigarettes per day for a year were included. The amount of cigarette smoking was based on total exposure measured by pack–years, which was the number of years of smoking multiplied by the number of packs of cigarettes smoked per day.

Patients with age ≥ 50 years old, equivalent spherical diopter of patients with ametropia ≤ 2.5 D, best-corrected visual acuity (BCVA) of at least 1.0 logarithm of the minimum angle of resolution and intraocular pressure (IOP) between 10–21 mmHg and axial length (AL) ≤ 25 mm were included. According to the AMD basic clinical classification proposed by Ferris FL [13], those with no drusen and no AMD pigmentary abnormality or those with only drupelets (small drusen ≤ 63 μm) and no AMD pigmentary abnormality were placed into the normal control group, whereas those with medium drusen (diameter = 64–125 μm) and no AMD pigmentary abnormality or those with large drusen (diameter > 125 μm) and/or any AMD pigmentary abnormality were placed into the dry AMD group. Participants with any ocular disease that prevents the examination of the cornea and retina, previous ocular surgery (including anti-VEGF), ocular or systemic disease, presence of choroidal neovascularization and/or any GA, any maculopathy secondary to causes other than AMD or previous history of conservative treatment of retina were excluded from the study. All subjects received blood pressure examination on the day of examination, in order to exclude the influence of systemic perfusion on regional retinal blood flow. The patients with blood pressure > 140/90 mmHg were not included.

### Ocular examinations

All participants underwent comprehensive ophthalmic examinations, including BCVA, slit lamp microscope, IOP measurement, AL measurement by using the IOL Master (Carl Zeiss, Dublin, CA), dilated fundus examination and colour fundus photographic examination (Kaidianrui, Wuhan, Hubei). The swept-source OCTA imaging (Optovue, Fremont, CA) was performed by the same senior ophthalmologist on the day at 9:00 am–12:00 nn to rule out the effect of the circadian rhythm of choroidal vessels. All subjects in the smoking group were prohibited from smoking 8 h before the examination to eliminate the acute choroidal effect caused by smoking.

### OCTA measurements

The angio retina 6.0 mm scan mode of the OCTA device with an 840 nm light source was chosen to capture a 6 mm × 6 mm area centred on the macula. The built-in software of OCTA can stratify the retina and choroid into SCP, DCP, outer retina and choroidal capillary layer automatically. Images with significant segmentation errors, severe atrophy involving the inner retinal layers or signal strength of 5 or less were excluded from the analysis. The vascular density was defined as the percentage of blood vessels in the 3 mm × 3 mm macular cube area centred on the fovea. The vessel density is the percentage of vessels and microvessels in the selected area. The software in OCTA can automatically measure the vessel densities of FAZ, FD, SCP, DCP and FRT in the fovea. In the dry AMD group, the diameter of the drusen was measured manually by a calliper in the machine’s built-in software.

### Statistical analysis

The IBM SPSS Statistics 26.0 was used for data analysis. Descriptive analysis was carried out for all parameters. The Shapiro–Wilk test was used to check the normal distribution of the variables. The independent samples t-test, repeated-measures analysis of variance (ANOVA) and Pearson’s correlation analysis were used for the statistical analysis of the data. When no indication of normal distribution after either Shapiro–Wilk tests or after Levene test for equality of variances was observed, the Mann–Whitney test, Kruskal–Wallis rank sum test and Spearman correlation analysis were used for the statistical analysis of the data. *P* < 0.05 was considered statistically significant.

## Results

### Demographics

A total of 134 study subjects, including normal control nonsmoking (49 cases, 49 eyes), normal control smoking (12 cases, 12 eyes), dry AMD nonsmoking (38 cases, 38 eyes) and dry AMD smoking (35 cases, 35 eyes) groups, were enrolled in this study. Age (*P* = 0.06) and pack–year history (*P* = 0.66) were not significantly different amongst the four groups. A significant difference in gender was observed amongst the groups (*P* < 0.01). In this study, all subjects in the smoking groups were male, which was related to the uneven gender distribution of the smoking group and was an uncontrollable factor (Table 1).

**Table 1:** Demographic and clinical data of each group

Variables	Control nonsmoking (*n* = 49)	Control smoking (*n* = 12)	Dry AMD nonsmoking (*n* = 38)	Dry AMD smoking (*n* = 35)	*P* value
Age (years), mean ± SD	63.39 ± 8.38	60.75 ± 7.11	66.97 ± 9.41	66.00 ± 7.67	0.06
Gender, male/female	17/32	12/0	12/26	35/0	< 0.01
Pack–year history, mean ± SD	0	35.79 ± 13.91	0	43.5 ± 31.61	0.66

*Abbreviations*: *AMD* Age-related macular degeneration

### Macular retina-related indices in four groups

**Fig. 1:**
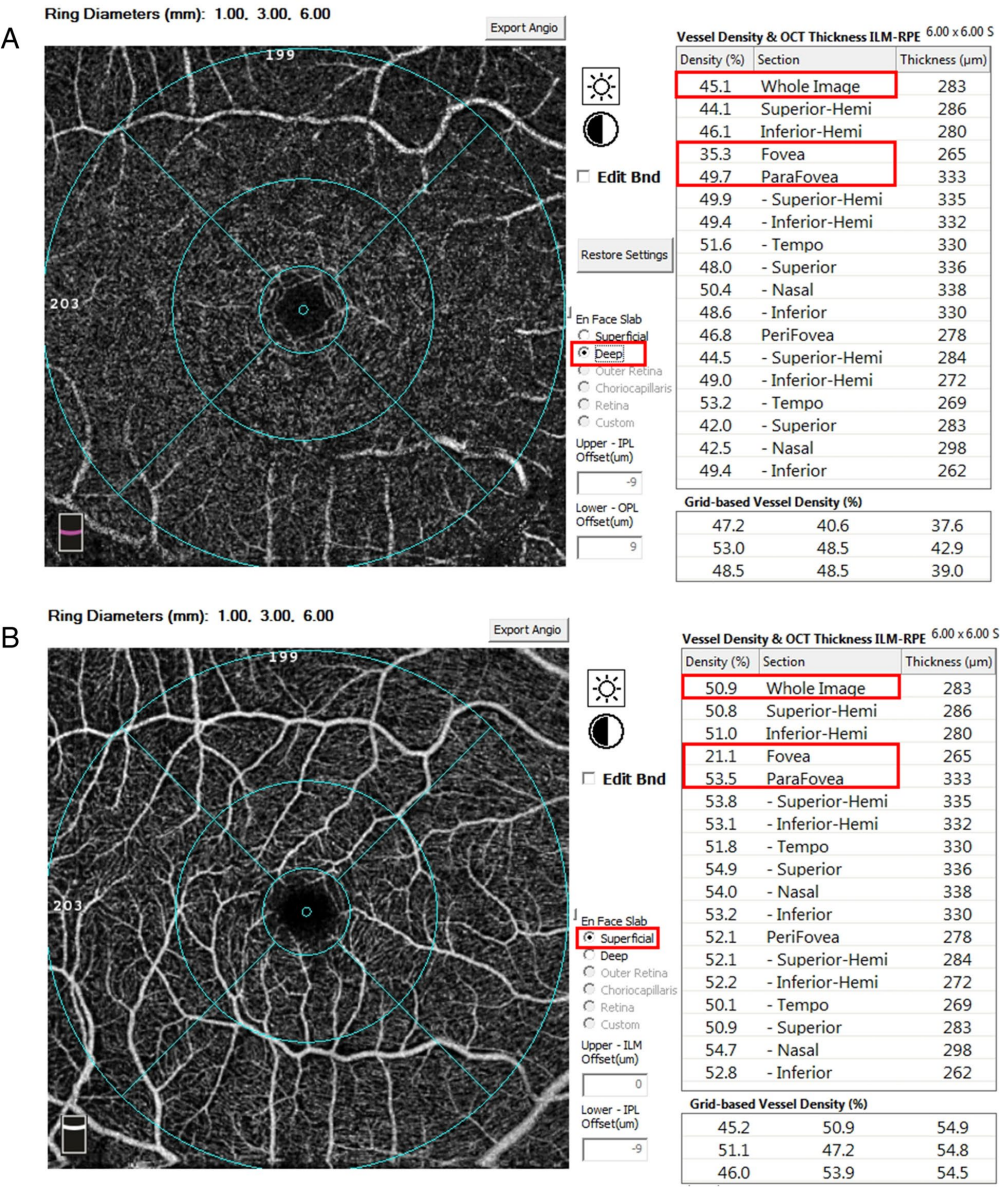
Calculation of capillary plexuses in normal control nonsmoking group with automated software (Optovue, Fremont, CA). Figure 1**A** represents the whole, foveal and parafoveal vessel density of deep capillary plexuses (DCP) in the normal control nonsmoking group measured by optical coherence tomography angiography (OCTA): the whole vessel density of DCP was 45.1%, the foveal vessel density of DCP was 35.3%, the parafoveal vessel density of DCP was 49.7%. Figure 1**B** represents the whole, foveal and parafoveal vessel density of superficial capillary plexuses (SCP) in the normal control nonsmoking group measured by optical coherence tomography angiography (OCTA): the whole vessel density of SCP was 50.9%, the foveal vessel density of SCP was 21.1%, the parafoveal vessel density of SCP was 53.5%

**Fig. 2:**
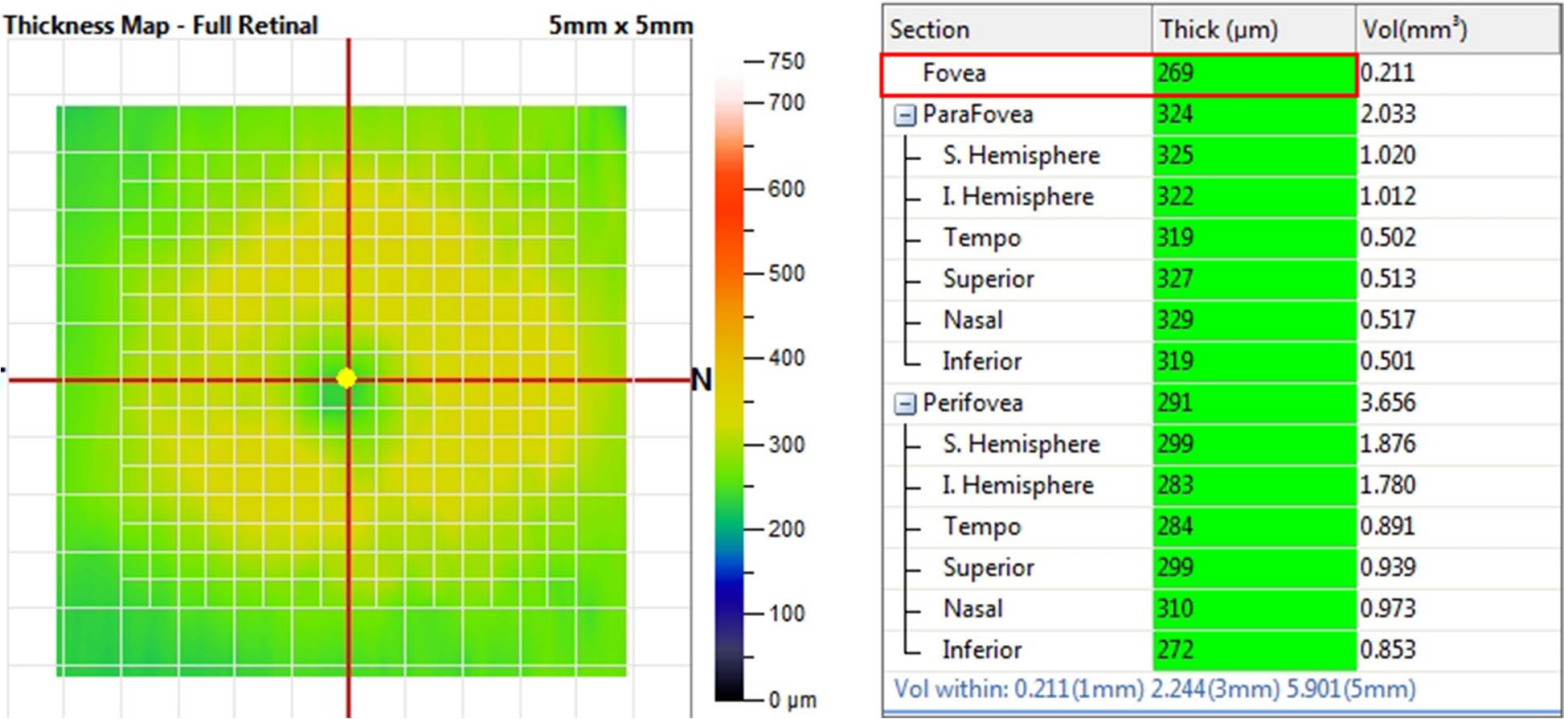
Calculation of full-layer of central fovea retinal thickness in normal control nonsmoking group with automated software (Optovue, Fremont, CA) The value of full-layer of central fovea retinal thickness (FRT) in the normal control nonsmoking group measured by optical coherence tomography angiography (OCTA) equals to 269

In the comparative analysis of the FAZ area, no statistically significant difference was observed amongst the four groups (*P* = 0.15, Table 2). The FD in the normal control nonsmoking group was significantly higher than that in the dry AMD nonsmoking group (*P* = 0.02). The whole, foveal and parafoveal vessel densities of SCP and DCP in the control nonsmoking group (Fig. 1) were all significantly higher than those in the dry AMD nonsmoking group (all *P* < 0.05), whereas the whole vessel density of SCP in the normal control smoking group was significantly higher than that in the dry AMD smoking group (*P* = 0.04). The foveal vessel density of SCP in the dry AMD smoking group was significantly higher than that in the dry AMD nonsmoking group (*P* < 0.01), and an increasing trend was observed in other blood flow indices but had no significant difference. The thickness values of the inner and full-layer FRT (Fig. 2) in the normal control nonsmoking group were significantly higher than those in the dry AMD nonsmoking group (all *P* < 0.01). Compared with that in the dry AMD smoking group, the thickness of the full-layer FRT in the normal control smoking group was significantly higher (*P* = 0.04).

**Table 2:** Statistical description of macular retina-related detection indices in each group

Variables	Control nonsmoking (*n* = 49)	Control smoking (*n* = 12)	Dry AMD nonsmoking (*n* = 38)	Dry AMD smoking (*n* = 35)	*P* value
FAZ (mm^2^), mean ± SD	0.33 ± 0.12	0.32 ± 0.12	0.41 ± 0.20	0.35 ± 0.13	0.15
FD (%), mean ± SD	50.56 ± 5.64^a^	49.11 ± 6.89^ab^	44.62 ± 9.65^b^	47.77 ± 7.35^ab^	0.02
SCP					
whole (%), mean ± SD	46.70 ± 4.64^a^	47.84 ± 4.77^a^	43.56 ± 5.26^b^	44.58 ± 4.02^b^	< 0.01
foveal (%), mean ± SD	16.82 ± 6.43^a^	18.48 ± 4.43^a^	12.52 ± 6.58^b^	15.83 ± 6.12^a^	< 0.01
parafoveal (%), mean ± SD	48.94 ± 5.54^a^	50.27 ± 5.84^a^	44.77 ± 5.36^b^	46.64 ± 5.30^ab^	< 0.01
DCP					
whole (%), mean ± SD	47.19 ± 6.41^a^	44.34 ± 7.83^ab^	42.95 ± 7.52^b^	44.46 ± 5.16^ab^	0.03
foveal (%), mean ± SD	30.77 ± 8.08	30.72 ± 5.77	26.81 ± 7.73	29.61 ± 8.75	0.13
parafoveal (%), mean ± SD	53.99 ± 4.86^a^	49.03 ± 8.24^ab^	49.86 ± 7.48^b^	50.13 ± 5.50^b^	< 0.01
full-layer FRT (mm), mean ± SD	252.35 ± 28.94^a^	260.17 ± 26.28^a^	236.68 ± 32.60^b^	229.74 ± 45.42^b^	< 0.01
inner FRT (mm), mean ± SD	67.80 ± 20.40^a^	69.83 ± 23.80^ab^	56.10 ± 18.76^b^	59.74 ± 15.15^ab^	< 0.01
outer FRT (mm), mean ± SD	184.55 ± 14.53^ab^	194.25 ± 13.57^a^	180.58 ± 27.97^b^	174.06 ± 36.86^ab^	0.03

*Abbreviations*: *FAZ* Foveal avascular zone, *FD* Foveal density in a 300 μm region around FAZ, *SCP* Superficial capillary plexuses, *DCP* Deep capillary plexuses, *FRT* Fovea retinal thickness. Different letters indicate that the difference is statistically significant, a > b

### Macular retina-related indices in dry AMD and control groups

The FD in the normal control group was significantly higher than that in the dry AMD group (*P* < 0.01). The whole, foveal and parafoveal vessel densities of SCP in the dry AMD group were significantly decreased compared with those in the control group (all *P* < 0.01). The whole (*P* = 0.01) and parafoveal (*P* < 0.01) vessel densities of DCP in the control group were significantly higher than those in the dry AMD group. The inner and outer and full-layer FRT in dry AMD eyes were thinner than those in control eyes (all *P* < 0.05, Table 3).

**Table 3:** Statistical description of macular retina-related indices in the normal control and dry AMD groups

Variables	Control (*n* = 61)	Dry AMD (*n* = 73)	*P* value
FAZ (mm^2^), mean ± SD	0.33 ± 0.12	0.38 ± 0.17	0.07
FD (%), mean ± SD	50.28 ± 5.88	46.13 ± 8.71	< 0.01
SCP			
whole (%), mean ± SD	46.93 ± 4.64	44.05 ± 4.71	< 0.01
foveal (%), mean ± SD	17.14 ± 6.10	14.11 ± 6.53	< 0.01
parafoveal (%), mean ± SD	49.20 ± 5.57	45.67 ± 5.38	< 0.01
DCP			
whole (%), mean ± SD	46.63 ± 6.74	43.67 ± 6.50	0.01
foveal (%), mean ± SD	30.76 ± 7.64	28.15 ± 8.30	0.06
parafoveal (%), mean ± SD	53.02 ± 5.94	49.99 ± 6.56	< 0.01
full-layer FRT (mm), mean ± SD	255.03 ± 30.38	232.26 ± 38.79	< 0.01
inner FRT (mm), mean ± SD	68.20 ± 20.91	57.85 ± 17.10	< 0.01
outer FRT (mm), mean ± SD	186.46 ± 14.76	177.45 ± 32.47	0.02

*Abbreviations*: *FAZ* Foveal avascular zone, *FD* Foveal density in a 300 μm region around FAZ, *SCP* Superficial capillary plexuses, *DCP* Deep capillary plexuses, *FRT* Fovea retinal thickness

### Macular retina-related indices in smoking and nonsmoking groups

The parafoveal vessel density of DCP in the nonsmoking group was significantly higher than that in the smoking group (*P* = 0.02). No statistical difference in other indices was observed between nonsmoking and smoking groups (all *P* > 0.05, Table 4).

**Table 4:** Statistical description of macular retina-related indices in the smoking and nonsmoking groups

Variables	Nonsmoking (*n* = 87)	Smoking (*n* = 47)	*P* value
FAZ (mm^2^), mean ± SD	0.37 ± 0.16	0.34 ± 0.13	0.51
FD (%), mean ± SD	47.97 ± 8.16	48.11 ± 7.18	0.88
SCP			
whole (%), mean ± SD	45.33 ± 5.14	45.41 ± 4.41	0.92
foveal (%), mean ± SD	14.94 ± 6.80	16.51 ± 5.81	0.18
parafoveal (%), mean ± SD	47.12 ± 5.81	47.56 ± 5.61	0.67
DCP			
whole (%), mean ± SD	45.34 ± 7.19	44.42 ± 5.86	0.46
foveal (%), mean ± SD	29.04 ± 8.13	29.90 ± 8.05	0.56
parafoveal (%), mean ± SD	52.19 ± 6.44	49.84 ± 6.23	0.02
full-layer FRT (mm), mean ± SD	244.72 ± 31.48	238.74 ± 45.36	0.37
inner FRT (mm), mean ± SD	62.69 ± 20.44	62.32 ± 18.02	0.79
outer FRT (mm), mean ± SD	182.82 ± 21.41	179.21 ± 33.58	0.71

*Abbreviations*: *FAZ* Foveal avascular zone, *FD* Foveal density in a 300 μm region around FAZ, *SCP* Superficial capillary plexuses, *DCP* Deep capillary plexuses, *FRT* Fovea retinal thickness

### Correlation analysis between pack–year history and macular retina-related indices

The pack–year history was negatively correlated with the parafoveal vessel density of DCP (*r* =  − 0.224, *P* < 0.01, Table 5).

**Table 5:** Correlation between pack–year history and macular retina-related detection indices

Pack–year history	FAZ (mm^2^)	FD (%)	SCP			DCP			FRT		
			whole (%)	foveal (%)	para- foveal (%)	whole (%)	foveal (%)	para- foveal (%)	full- layer (mm)	inner (mm)	outer (mm)
*r*	-0.055	-0.023	-0.053	0.096	0.016	-0.109	0.039	-0.224^**^	-0.033	0.030	0.021
*P* value	0.529	0.318	0.541	0.270	0.856	0.210	0.658	0.009	0.705	0.732	0.813

*Abbreviations*: *FAZ* Foveal avascular zone, *FD* Foveal density in a 300 μm region around FAZ, *SCP* Superficial capillary plexuses, *DCP* Deep capillary plexuses, *FRT* Fovea retinal thickness. *, **, ***indicate that they are statistically significant on 10%, 5%, 1% respectively; *r* is the correlation coefficient, *r* > 0 means there is a positive correlation between the two variables, *r* < 0 means that the two variables are negatively correlated, and *r* = 0 means that the two variables are zero related. | *r* | the closer to 1, the higher the correlation between the two variables

### Correlation analysis between FRT and vessel densities of SCP and DCP

The inner FRT was positively correlated with the foveal vessel density of SCP (*r* =  + 0.223, *P* < 0.01). The outer FRT was positively correlated with the foveal vessel density of DCP (*r* =  + 0.361, *P* < 0.001, Table 6).

**Table 6:** Correlation between inner FRT and SCP, outer FRT and DCP

	SCP and inner FRT (mm)			DCP and outer FRT (mm)		
	whole (%)	foveal (%)	parafoveal (%)	whole (%)	foveal (%)	parafoveal (%)
*r*	0.117	0.223^**^	0.036	0.033	0.361^**^	-0.082
*P* value	0.180	0.009	0.677	0.708	< 0.001	0.346

*Abbreviations*: *SCP* Superficial capillary plexuses, *DCP* Deep capillary plexuses, *FRT* Fovea retinal thickness. ^*^, ^**^, ^***^indicate that they are statistically significant on 10%, 5%, 1% respectively; *r* is the correlation coefficient, *r* > 0 means there is a positive correlation between the two variables, *r* < 0 means that the two variables are negatively correlated, and *r* = 0 means that the two variables are zero related. | *r* | the closer to 1, the higher the correlation between the two variables

## Discussion

OCTA is a noninvasive procedure and can be depth-segmented to afford 3D volumetric scans. By using motion contrast to acquire images, the vasculature of the retina and choroid can be visualised to display structural and blood flow information and is used as an imaging method for evaluating various retinal diseases [14]. Thus far, no study has evaluated the detailed characteristics of retinal vasculature in smoking patients with early and intermediate dry AMD through an imaging approach.

In this cross-sectional control study, we investigate retinal vessel features and other retina-related indices in patients affected by early and intermediate dry AMD by using OCTA. Overall, we found that retinal vessels and retina-related indices are altered in these patients. The whole, foveal and parafoveal vessel densities of SCP and the whole and parafoveal vessel densities of DCP in patients with early and intermediate dry AMD are all significantly lower than those in normal controls. This difference also occurs in the dry AMD and control nonsmoking subgroups, and the sensitivity of SCP vessel density is higher than that of DCP vessel density. This finding may be because OCTA focuses on the superficial retina to avoid the influence of attenuation and artifacts of vitreous opacity or other AMD pathologically related signals and the shadow effect of deep-layer vessel density in the retina [15, 16]. We speculate that the loss of retinal vessels in patients with early and intermediate dry AMD may stimulate the ischaemia mechanism of retina. The loss of retinal cells can be caused by this mechanism, which may indicate that the changes in retinal vessels play a certain role in the dry AMD pathogenesis. In a previous study, the change in the vessel density of SCP has been well tested by OCTA [17, 18]. Mastropasqua et al. [19] found a significant reduction in superficial and deep retinal vessel densities in patients with AMD compared with those in healthy controls. Our results are consistent with those in previous studies. We also found that the value of foveal vessel density of SCP in the dry AMD smoking group is significantly higher than that in the dry AMD nonsmoking group. Moreover, although no significant difference is observed in the other indices of SCP and DCP, an increasing trend is found in the AMD smoking group compared with that in the AMD nonsmoking group. At present, no relevant literature is available to explain this phenomenon. We speculate that this finding may be due to a compensatory increase in retinal blood supply with early and intermediate dry AMD long-term smokers. Chronic smoking and dry AMD can cause the loss of choroidal capillaries and ischaemia, which may lead to the compensatory increase in retinal blood. In the normal control group, the SCP blood density of the smoking subgroup also has an increasing trend compared with that of the nonsmoking subgroup, but no statistical difference is observed. However, for patients with AMD, the compensatory effects are more pronounced in the smoking subgroup than in the nonsmoking subgroup. To a certain extent, the mechanism of the compensatory increase in blood flow caused by smoking has weakened the difference in blood flow changes in dry AMD smokers with control smokers. However, this finding is only our conjecture, and we need to combine the analysis of a large number of clinical samples and the histopathological study of retina and choroid for verification.

FAZ shows as a clear round or oval nonvascular signal area in OCTA and is often used as a quantitative analysis index to measure macular ischaemia, which is related to retinal function damage. In this study, we found no significant change in FAZ in normal control group and dry AMD group and their subgroups. Many authors revealed no significant difference in FAZ and shape in between the early and intermediate dry AMD and control groups [20, 21]. The results of our study are consistent with those of previous studies, suggesting that the perfusion of central vessels in the fovea may not play a role in the pathogenesis of dry AMD. This finding is different from the results of FAZ in retinal vascular diseases, such as diabetic retinopathy or retinal vein occlusion, the severity of which is related to the enlargement of FAZ [22]. In addition, we found that the value of FD in the normal nonsmoking group is significantly higher than that in the dry AMD nonsmoking group. In the current research, articles about the relationship between FD and dry AMD remain lacking.

Smoking is a major and changeable risk factor for AMD, which poses a serious threat to the normal function of human vascular system. Long-term smoking can cause irreversible damage to blood vessels by increasing the flow resistance of terminal blood vessels and reducing blood flow. Mechanisms include direct toxic effects on the endothelial cells of ocular blood vessels, damage of the vascular structure and destruction of vascular function [23–25]. The components in cigarette smoke have a direct toxic effect on the endothelium, and nicotine may cause structural damage in vitro and in vivo [23, 26]. The differences between OCTA systems and measurement methods must be considered when comparing different research literature. At the same time, the parafoveal vessel density of DCP in the smoking group is significantly lower compared with that in the control group. This result is consistent with that of a previous study, suggesting that smoking may cause a local ischaemic microenvironment, which may be a risk factor for the development of choroidal neovascularisation in smokers [27]. Given the multiple properties, including the distance from the large arterioles, the complex vascular structure and the high metabolic activity [28], DCP is susceptible to oxidative damage and poor perfusion caused by smoking [29]. Our study also showed that the pack–year history is negatively correlated with the parafoveal vessel density of DCP. We can infer that with prolonged smoking, the vessel density of DCP decreases. This finding explains that the chronic effects of smoking damage microvessels continuously, highlighting the importance of quitting smoking for health.

In accordance with our results, the inner and full-layer FRT of dry AMD group are thinner than that of the normal group. The reason may be the decrease in the retinal vessel density of patients with dry AMD and the ischaemia mechanism in the retina, leading to decreased intraretinal cells. Rogala et al. [30] showed inner retinal layer thinning in patients with dry AMD. Our results are consistent with those of previous studies. At the same time, the inner FRT is positively correlated with the foveal vessel density of SCP, and the outer FRT is positively correlated with the foveal vessel density of DCP. Therefore, we speculate that dry AMD may lead to changes in the structure and blood flow of the retina first and that the microvascular insult and thinning of retinal structure are mutual cause and effect. The alteration in retinal structural and microvascular properties may further elucidate the progress of dry AMD. Next, we can refine the staging and observe the sequence of changes in the retinal structure and blood flow as dry AMD progresses. The combination of these two indicators can be used to provide reference clinical indicators for the research pathogenesis on dry AMD. According to previous data, an evident correlation between smoking and FRT is not observed [12, 31, 32]. In the present study, we found no correlation between pack–year history and FRT and no significant change in FRT in groups of smoking and nonsmoking. These findings are consistent with those in previous data. However, relevant research to further prove the relationship between FRT and dry AMD remains lacking.

Quantitative fundus autofluorescence (qAF) of dry AMD found a decrease from normal to early to late AMD, suggesting that loss of lipofuscin fluorophores signifies AMD progression [33]. Full-field electroretinography (ffERG) suggests a more diminished overall response compared late AMD to healthy controls [34]. In the next step, we can use ffERG, qAF and OCTA to further study the effect of smoking on the retina of late AMD. Also we can assisted by artificial intelligence (AI) to analyse the relationship between the progress of dry AMD and the detection index of the retina. Remarkable differences in the gender of the included researchers are observed because the gender distribution of smoking groups is uneven, which is an irreconcilable contradiction. Thus, we should expand the sample size of each group to improve the study. Furthermore, large studies that investigate the chronic effects of smoking on retinal microcirculation in the macula may help explain the role of smoking as a risk factor on systemic vascular diseases.

## Conclusions

In conclusion, FD, SCP, DCP and FRT are sensitive indices for the detection of early and intermediate dry AMD. DCP is a sensitive indicator that reflects the effects of smoking on the retina. Considerable changes are observed in retinal vessels, suggesting that dry AMD may affect the retinal tissue to a certain extent.

## Data Availability

The data that support the findings of this study are available on request from the corresponding author, Yan Li. The data are not publicly available due to their containing information that could compromise the privacy of research participants.

## Abbreviations

OCTA: Optical coherence tomography angiography
AMD: Age-related macular degeneration
FAZ: Foveal avascular zone
FD: Foveal density
SCP: Superficial capillary plexuses
DCP: Deep capillary plexuses
FRT: Fovea retinal thickness
RPE: Retinal pigment epithelium
GA: Geographic atrophy
VEGF: Vascular endothelial growth factor
BCVA: Best-corrected visual acuity
IOP: Intraocular pressure
AL: Axial length
qAF: Quantitative fundus autofluorescence
ffERG: Full-field electroretinography
AI: Artificial intelligence
